# Testing predictive models of positive and negative affect with psychosocial, acculturation, and coping variables in a multiethnic undergraduate sample

**DOI:** 10.1186/2193-1801-3-119

**Published:** 2014-03-01

**Authors:** Ben CH Kuo, Catherine T Kwantes

**Affiliations:** University of Windsor, Windsor, Canada; Department of Psychology, University of Windsor, 401 Sunset Ave, Chrysler Hall South, Windsor, Ontario N9B 3P4 Canada

**Keywords:** Positive affect, Negative affect, Acculturation, Cultural coping, Religious coping

## Abstract

Despite the prevalence and popularity of research on positive and negative affect within the field of psychology, there is currently little research on affect involving the examination of cultural variables and with participants of diverse cultural and ethnic backgrounds. To the authors’ knowledge, currently no empirical studies have comprehensively examined predictive models of positive and negative affect based specifically on multiple psychosocial, acculturation, and coping variables as predictors with any sample populations. Therefore, the purpose of the present study was to test the predictive power of perceived stress, social support, bidirectional acculturation (i.e., Canadian acculturation and heritage acculturation), religious coping and cultural coping (i.e., collective, avoidance, and engagement coping) in explaining positive and negative affect in a multiethnic sample of 301 undergraduate students in Canada. Two hierarchal multiple regressions were conducted, one for each affect as the dependent variable, with the above described predictors. The results supported the hypotheses and showed the two overall models to be significant in predicting affect of both kinds. Specifically, a higher level of positive affect was predicted by a lower level of perceived stress, less use of religious coping, and more use of engagement coping in dealing with stress by the participants. Higher level of negative affect, however, was predicted by a higher level of perceived stress and more use of avoidance coping in responding to stress. The current findings highlight the value and relevance of empirically examining the stress-coping-adaptation experiences of diverse populations from an affective conceptual framework, particularly with the inclusion of positive affect. Implications and recommendations for advancing future research and theoretical works in this area are considered and presented.

Over the last three decades there has been significant growth in our understanding of the associations among coping, emotion, and adaptation with respect to individuals’ responses to life changes (e.g., chronic illnesses, death, loss, etc.) both conceptually and empirically (Aldwin 2007; Folkman and Moskowitz 2000; Watson et al. 1988). The development of this body of research is most evident in the increasing number of scholarly works focusing on the co-existence of ‘positive affect’ and ‘negative affect,’ the correlates associated with each of these affects, and the consequences these affective experiences have on individuals’ personal well-being and adjustment (e.g., Diener and Emmons 1985; Moskowitz et al. 2012). The impetus to study positive affect, in particular, has been heightened by the prominence of the positive psychology movement in recent decades which underlines the benefit of studying the healthy and salubrious aspects of human experiences (Folkman and Moskowitz 2003). As a result, many contemporary theories and research on stress, coping, and adaptation regard individuals’ affective and emotion experiences as central elements in their conceptual schemas (Folkman 2008).

Acculturation characterizes a particular instance of stress and coping that involves individuals and groups coping with, and adjusting to, a new environment (Berry 1997), including members of immigrant groups and ethnic minority groups (Kuo 2014). Prominent acculturation scholars John Berry (1997,2006) and Colleen Ward (2001) have noted that the underpinning principles of acculturation theory are deeply rooted in the classic, person-environment transactional theory of stress-coping-adaptation by Lazarus and Folkman (1984). A recent comprehensive review by Kuo (2014) further marshals evidence clearly pointing to the pervasive and profound influences of the stress-coping-adaptation paradigm on contemporary theories of acculturation and cultural adjustment.

Consider the above, however, current research examining the link between acculturation and affects has been quite limited, as most acculturation research has focused adaptation in terms of measures of psychological distress or symptomologies (i.e., depression or anxiety) or sociocultural patterns (Ward 2001) and has not specifically included affective outcomes. Some recent empirical works have just begun inquiring into this relationship, but the findings are quite preliminary (see examples, Paukert et al. 2006; Pan et al. 2008; Pan and Wong 2011). As well, there has been little empirical knowledge about the protective antecedents associated with positive and negative affect among culturally and ethnically diverse populations. Again, this is because research on affective well-being among ethnic minorities is at the present simply very scarce (Villodas et al. 2011).

Despite the above observations, a close look at the extant cross-cultural and multicultural psychological literature reveals that a number of critical psychosocial and cultural variables have been identified and shown to be predictive of the well-being and the adaptation of culturally diverse individuals. These variables have included acculturation and enculturation (Yoon et al. 2012; Nguyen and Benet-Martinez 2013), social support (Kim et al. 2008), diverse cultural coping behaviors (Kuo 2011; Kuo et al. 2006), and religious coping behaviors (Tarakeshwar et al. 2003), to name a few. While these variables have not been simultaneously examined in predicting positive and negative affect, the cumulative literature offers some clues to the potential of these factors in helping us understand the qualities and the correlates associated with affect among culturally diverse populations.

To date no empirically published studies known to the authors have systematically tested comprehensive predictive models of positive and negative affect on the basis of previous theories and included the above described psychosocial, acculturation and coping variables as predictors. In addition, some scholars have called for more research on positive and negative affect with multiculturally diverse populations because such empirical work is currently lacking (Pan 2011; Villodas et al. 2011). Therefore, the purpose of the present study was to explore and test two predictor models of positive and negative affect with perceived stress, social support, bidirectional acculturation (i.e., Canadian acculturation and heritage acculturation), religious coping and cultural coping (i.e., collective, avoidance, and engagement coping) as predictors in a sample of multiethnic university students in Canada. Informed by the theories of stress-coping-emotion and acculturation and guided by Fitzell and Pakenham’s (2010) psychosocial stress-coping model of positive and negative adjustment, the current study aimed to assess the collective (additive) as well as the individual contributions of these factors in predicting affect in the current sample.

## Theoretical frameworks

### Stress-coping-emotion theories

Lazarus and Folkman's (1984) Cognitive Theory of Stress and Coping proposes that emotions are the corollaries of an individual’s appraisal of a stressful situation. These affective responses then prompt individuals to decide what type of coping is to be engaged in as a response to the stressor. Typically, it is hypothesized that problem-focused coping strategies would lead to more positive affect, whereas emotion-focused and avoidance coping strategies would lead to more negative affect (Folkman 1984). Accordingly positive and negative affect have been shown to be two independent sets of emotional experiences and expression that co-exist in individuals – known as the “Two-factor’ theory of affect (Diener and Emmons 1985; Villodas et al. 2011). This orthogonal view of positive and negative affect is well illustrated by Watson et al.'s (1988) instrument development study of the Positive and Negative Schedule (PANAS) – a widely researched measure of affects that was also used in the current study. These researchers defined high positive affect as a state of “high energy, full concentration, and pleasurable engagement” (p. 1063; Watson et al. 1988) and are characterized by enthusiastic, active and alert feelings. Negative affect, on the other hand, is defined as “subjective distress and unpleasant engagement that subsumes a variety of averse mood states” (p.1063; Watson et al. 1988) and include feelings such as anger, contempt, disgust, guilt, fear, and nervousness.

With respect to coping specifically, there has been increasing conceptual and empirical evidence pointing to the effects of culture in shaping coping patterns and stress-coping processes particularly among members of culturally diverse groups (Kuo 2011,2014; Wong and Wong 2006). Recent comprehensive reviews of the literature have shown that diversity in preferences for different coping behaviors among racial, ethnic, and national groups clearly exists and that these cross-cultural differences in coping patterns are related to cultural dimensions, such as collectivism-individualism, interdependent-independent self construals, and acculturation (Kuo 2011,2013). This emerging body of cultural coping literature stresses the additional importance of examining the cultural aspects of coping overlooked by the prevailing, individualism-based, Western stress-coping theories and research in the extant literature (Wong and Wong 2006), such as the phenomenon of ‘collective coping’ – stress responses that derive from a person’s or a group’s collectivism value or orientation (Kuo 2013). In view of this emerging literature on culture and coping, it is therefore critical to assess culture’s role in studying the coping experiences of individuals with diverse ethnic and cultural backgrounds, as was the case for the sample of the current study. For this reason, the Cross-Cultural Coping Scale (CCCS: Kuo et al. 2006), a culturally-derived coping measure, was employed to assess the coping behaviors of the current sample. Specific information about the CCCS is described in the Measures section later in the paper.

### Psychosocial predictor model of affects

Currently limited research or conceptual models have addressed the concurrent effects of multiple psychosocial antecedents of positive and negative affect. One rare exception is Fitzell and Pakenham’s (2010) psychosocial stress-coping model of positive and negative adjustment. The authors identified a constellation of predictive factors for positive and negative adjustment. They then tested the model based on a sample of 622 caregivers of patients diagnosed with colorectal cancer in Australia. In this model Fitzell and Pakenham posited that stress appraisal, social support, and coping would be predictive of caregivers’ positive adjustment, as measured by positive affect and other adjustment indicators such as life satisfaction, and of caregivers' negative adjustment, as measured by psychological distress. The results of the regression analyses after controlling for the caregivers’ demographics showed that perceived stress, coping strategies, and social support were all significant predictors of both positive and negative affect in the expected directions. This present study extended and expanded on Fitzell and Pakenham’s original psychosocial model by including additional cultural variables (i.e., Canadian acculturation, heritage acculturation and collective coping behaviors) and by testing the model with an ethnically diverse sample.

### Acculturation theory

The seminal acculturation theory by John Berry (1997,2006) represents the most influential and the most widely cited psychological theory of cultural adaptation. This acculturation theory is derived from Lazarus and Folkman’s (1984) early research on transactional theory of stress and coping (Berry 2006). At the individual level of acculturation, Berry’s theory posits that multiple ‘moderating factors’ *prior to* and *during* acculturation can act to facilitate or impede on the quality of an acculturating person’s long term cultural adaptation in the host society (Berry 1997). More specifically, Berry hypothesized that moderating variables associated with the individual *prior to* acculturation, such as age, gender, education, religion and spirituality, and personality, interact with moderating variables *during* acculturation, such as acculturation strategies, coping strategies and resources, social support, etc. It is therefore conceptually germane that stress, coping, social support, religious/spiritual orientation, and acculturation are integral and interacting elements that contribute to the long term adaptation of individuals within this classic acculturation framework. These moderating factors are examined in the current study as potential predictors for affective outcomes. Specifically, acculturation is conceptualized and measured based on the bidirectional model of acculturation, assessing both participants’ cultural identification with the mainstream Canadian culture as well as with their heritage cultures (or enculturation) (Kuo 2010; Ryder et al. 2000).

## Research on factors predicting positive and negative affect

### Psychosocial variables

Previous research has provided some clues to the nature and direction of the relationships between affects and the psychosocial and cultural variables examined in the present research. In terms of negative affect, higher levels of life stress/strain, higher levels of avoidance and emotion-focused coping, lower levels of daily spiritual experience, lower levels of social support, and lower levels of coping have been found to be associated with higher levels of negative affect across a number of studies, including a community-based survey of women in Canada (Nelson 1990), a health survey of adult population in the U.S. (Whitehead and Bergeman 2011), and an earlier research by Folkman (1984). Furthermore, in a recent model testing study of 623 undergraduate students in Canada, Green et al. (2012) found a significant direct positive relationship between participants’ negative affect and stress. In the same study negative affect was, however, not related to the participants’ social support as measured either by support network size or support satisfaction.

On the other hand, lower levels of life stress/strain, higher levels of daily spiritual experience, higher levels of social support, and more frequent use of proactive and problem-focus coping behaviors have been found to predict higher levels of positive affect in several empirical studies (Folkman 1984; Whitehead and Bergeman 2011). In the model-testing study cited above by Green et al. (2012), it was further revealed that positive affect was significantly related to undergraduate participants’ support network size or satisfaction, though no relationships between participants’ reported level of stress and positive affect were found. In the study by Fitzell and Pakenham (2010) of caregivers of cancer patients mentioned earlier, social support and coping were found to be significant positive predictors of positive affect but their perception of stress was a negative predictor of positive affect.

### Acculturation-related variables

The authors’ literature review on *PsychInfo* yielded only a handful of published studies to have attempted to address the link between acculturation and affects. Pan and her colleagues published a series of articles on cross-cultural comparative study of adaptation of Chinese internationals students in Australia and Hong Kong using negative and positive affect as measures of outcome (e.g., Pan 2011; Pan and Wong 2011; Pan et al. 2008). As an example, in one study assessing positive affect among Chinese international students in Australia (N = 440) and Hong Kong (N = 227), Pan et al. (2008) found that positive affect was negatively predicted by acculturative stress due to academic work and social interaction in the host culture in the Hong Kong sample, whereas in the Australian sample, it was predicted by acculturative stress resulting only from social interaction.

In yet another more recent research reported by Pan and her colleagues, acculturative stressors and acculturative strategies were examined as predictors of negative affect in samples similar to the one described above (Pan and Wong 2011). “Marginalization’ acculturation strategy reported by Chinese international students in both Hong Kong and Australia predicted more negative affect, whereas “assimilation’ acculturation strategy reported by Chinese international students in Australia predicted less negative affect. Moreover, acculturative stress resulting from academic work positively predicted negative affect in both sample, but acculturative stress resulting from cultural differences positively predicted negative affect in the Hong Kong sample only. These differential predictive patterns of negative and positive affect related to acculturation are noted in other studies as well. For example, in a study of first-year ethnic minority university students in the U.S., it was found that participants’ acculturative stress was significantly related to negative affect, but not to positive affect (Paukert et al. 2006). Given these divergent findings and the scarcity of research on acculturation and affect, more research is clearly needed.

## The present study

The present study intended to address the following research question: *“To what extent do perceived stress, social support, Canadian acculturation, heritage acculturation, religious coping, collective coping, avoidance coping, and engagement coping predict and explain positive and negative affect, respectively, in the current multiethnic undergraduate sample in Canada?”* Two hypotheses were put forth to help address this research question and were tested with hierarchical multiple regressions.

### Hypothesis #1

It is posited that perceived stress, social support, Canadian acculturation, heritage acculturation, religious coping, collective coping, avoidance coping and engagement coping will significantly predict positive affect in the current sample. Specifically, it is hypothesized that more perceived stress, religious coping and avoidance coping would predict less positive affect, while more social support, Canadian acculturation, heritage acculturation, collective coping and engagement coping would predict more positive affect in the sample.

### Hypothesis #2

It is posited that perceived stress, social support, Canadian acculturation, heritage acculturation, religious coping, collective coping, avoidance coping and engagement coping will significantly predict negative affect in the current sample. Specifically, it is expected that more perceived stress, avoidance coping, and religious coping would predict greater negative affect, while more social support, Canadian acculturation, heritage acculturation, collective coping and engagement coping would predict less negative affect in the present student sample.

## Methods

### Procedure

A total of 301 participants were recruited through two separate methods with the aim to maximize the ethnic diversity of the sample: a) the participant pool system at the Department of Psychology at the University of Windsor, Canada, where this study was conducted; and b) various ethnic student organizations and the International Student Center on the university campus. With the former approach, participating students in the study were compensated with credit points assigned to their psychology course grades. For the latter group, for compensation participants were offered a chance to win one of five $10 gift certificates by entering their names into a draw. Prior to participating in the research the participants were presented with an informed consent form with an explanation about the purposes of the research and their rights according to the ethical procedures specified and approved by the University of Windsor’s Research Ethics Board. All participants signed the informed consent before they proceed to complete a paper-and-pencil questionnaire in the first author’s on-campus research lab.

Among the 301 undergraduate participants 229 were women and 72 were men, with a mean age of 22.8 (*SD* = 2.99). The significantly greater numbers of female participants (76%) than male participants (24%) in the sample likely reflected the higher proportion of women enrolling in psychology majors and courses than men. In terms of cultural characteristics, the sample was quite diverse. The sample comprised of 29.83% White/Europeans (*N*=88), 19.32% South Asians (*N*=57), 16.16% East Asians (*N*=49), 14.24% Blacks/Africans (*N*=42), 13.22% Middle Easterners (*N*=39) and 6.78% of those who identified Latinos/Latinas, Native Canadians, and mixed racial/ethnic backgrounds (*N*=20). With respect to immigrant status, the majority (79.4%) reported to be Canadian citizens, 14.3% reported to be international students, 5.6% reported to be landed immigrants, and 0.7% reported to be refugees. About one third of the sample identified themselves as first generation (32.9%) and second generation (32.6%) immigrant, respectively. A fifth of the participants identified as third or later generation (19.9%), and 14.6% identified as first generation international students.

### Measures

The description of all of the measures used to assess variables in the study is presented below. Means, standard deviations, and Cronbach alphas for all scales may be found in Table 1.

**Table 1:** **Means, SD, alphas of all variables**

Variable	Mean	SD	Alpha
Perceived stress	2.2	0.78	.79
Canadian acculturation	6.6	1.23	.85
Heritage acculturation	6.6	1.53	.89
Social support	5.4	1.25	.93
Religious coping	2.1	0.62	.78
Collective coping	3.7	0.84	.75
Avoidance coping	3.1	0.70	.70
Engagement coping	4.7	0.66	.77
Positive affect	3.3	0.79	.89
Negative affect	2.2	0.73	.85

*Perceived Stress Scale- 10 Item* (PSS: Cohen et al. 1983): The 10-item PSS is a self-report scale that assesses respondents’ perception of the degree of unpredictability and stress in their life during the past month. The scale was reported to have demonstrated favorable psychometric properties with high internal consistency across three college student samples (dormitory residents, psychology students, and student participants in a smoking-cessation program) in the original test development study (Cohen et al. 1983). Respondents rate their levels of stress on a 5-point Likert scale, with 0 = *never* to 4 = *very often*.

*Vancouver Index of Acculturation* (VIA: Ryder et al. 2000). The VIA is bidirectional measure of acculturation which assesses respondents’ cultural identification with the mainstream, dominant culture (i.e., Canadian culture) and with their heritage culture (i.e., the culture of birthplace or original ethnic culture) independently on two separate subscales. The original VIA consists of 20 items with half of the items assessing the mainstream subscale and the other half the heritage subscale. The two sets of items are parallel in their contents. Participants respond to the VIA on a 7-point Likert scale with 1 = *very strongly disagree* and 7 = *very strongly agree.* In the present study the score for the former was named ‘Canadian Acculturation’ and the latter ‘Heritage Acculturation’ in the analyses.

*Multidimensional Scale of Perceived Social Support* (MSPSS: Zimet et al. 1988). The 12-item MSPSS is a measure of individuals’ perception of interpersonal support and resources from: family, friends, and significant others, on a 7-point Likert scale with 1 = *very strongly disagree* and 7 = *very strongly agree.* The measure has sound psychometric properties with the internal consistency alphas for the subscales and the overall scale ranged from .85 to .91 and test-retest reliability ranged from .72 to .85 in the original test development study.

*Cross-Cultural Coping Scale* (CCCS: Kuo et al. 2006). The CCCS is a 26-item, scenario-based measure of coping behaviors developed and designed to assess both the collectivistic and individualistic aspects of stress responses (Kuo et al. 2006). The scale was originally tested with a sample of Chinese Canadian adolescents with varying degrees of acculturation levels (Kuo et al. 2006), but has subsequently been adopted in several research studies with culturally and developmentally diverse samples (e.g., Kuo and Gingrich 2004; Wester et al. 2006). The scale is comprised of three subscales: Collective Coping, Avoidance Coping, and Engagement Coping. The scale has demonstrated favorable psychometric properties in terms of internal consistency reliabilities of the subscales and criterion validity with acculturation levels and interdependent-independent self construals as reported in the original instrument development and validation study (Kuo et al. 2006). In the current study, the researchers used a hypothetical stress vignette based on a common stressful scenario undergraduate students often face, with respect to academic failure or poor academic performance. The scenario depicts a student’s depressive symptoms (e.g., sadness, hopelessness, problem with sleep and appetite, etc.) resulting from having difficulties with school works and performances and questioning of his/her status at the university and future career. The participants responded on how they would cope in such a scenario on each of the CCCS items on a 6-point Likert, with 1 = *A very inaccurate description of what I would do* and 6 = *A very accurate description of what I would do*.

*Brief RCOPE* (Pargament et al. 2000). Brief RCOPE is a 10-item measure that asks participants to think about major problems in their life and how they try to cope with it in relation to God. The items are scored on a Likert scale of 1 = *A great deal*, 2 = *Quite a bit*, 3 = *Somewhat*, 4 = *Not at all*. Items 1 to 5 on this measure are positive coping items, and items 6 to 10 are negative coping items. The scores of the latter items were reversed and combined with the scores of the former to form a single composite score of Religious coping in the analyses in the present study.

*Positive and Negative Affect Scale* (PANAS: Watson et al. 1988). The 20-item PANAS is a self-report measure designed to assess respondents’ positive affect (10 items) and negative affect (10 items). When tested with a sample of university students and community-based adults in the instrument development study, the internal consistency was .89 for positive affect and .85 for negative affect at the moment of responding to the measure. The 8-week test-retest reliability was .68 for positive affect and .71 for negative affect in general for the participants (Watson et al. 1988). The respondent is asked to indicate how she/he is experiencing each of the listed emotions currently based on a 5-point Likert scale with 1 = *Very slightly or not at all*, 2 = *Little*, 3 = *Moderately*, 4 = *Quite a bit*, 5 = *Extremely*. In a recent study by Villodas et al. (2011) the PANAS was found to demonstrate good reliabilities and convergent and discriminant validity with a sample of ethnically diverse adolescents in the U.S. comprised of Hispanic, Asian, African American, Native, Middle Eastern, and biracial youth.

### Framework for regressional analyses

Two separate hierarchical regression analyses with an identical set of predictors were conducted, one model predicting positive affect and the other negative affect. Given previous relationships found in the literature, participants’ levels of perceived stress, social support, and Canadian acculturation and heritage acculturation were controlled for by entering these variables in the first step of the regression. Religious coping, collective coping, avoidance coping, and engagement coping were entered into the second step of the equation.

## Results

### Correlations among variables

The correlations among the variables examined in the study are presented in Table 2. The correlation matrix shows that a number of variables are significantly related to positive and negative affect respectively. Positive affect was found to positively correlated with participants’ scores on social support, collective coping, and engagement coping, but negatively correlated with scores on perceived stress, religious coping and avoidance coping. On the other hand, negative affect was found to positively correlated with participants’ scores on perceived stress and avoidance coping, but negatively correlated with scores on engagement coping. In addition, positive affect and negative affect were significantly correlated with each other in a negative direction (*r* = −.27).

**Table 2:** **Correlations among variables in the study**

	HA	CA	SS	PS	RC	CC	AC	EC	NegAff	PosAff
HA		.03	.02	.05	-.16**	.36***	.04	.00	.08	.02
CA			.03	.04	.144*	-.07	-.02	.14*	.03	.06
SS				-.24***	-.09	.28***	-.11	.21***	-.11	.26***
PS					.06	-.14*	.30***	-.40***	.59***	-.49***
RC						-.21***	.14*	-.03	.04	-.25***
CC							-.03	.24***	-.03	.17**
AC								-.21***	.33***	-.26***
EC									-.26***	.44***
NegAff										-.27***
PosAff										

Note: *p < .05, **p < .01,***p < .001; HA = Heritage Acculturation; CA = Canadian Acculturation; SS = Social Support; PS = Perceived Stress; RC = Religious Coping; CC = Collective Coping; AC = Avoidance Coping; EC = Engagement Coping; NegAff = Negative Affect; PosAff = Positive Affect.

### Hypothesis 1 (regression model for positive affect)

The amount of perceived stress, social support, and both the degree of heritage acculturation and Canadian acculturation significantly predicted positive affect in the first step of the hierarchical regression, *F*(4, 292) = 26.84, *p* < .001, with the model accounting for 52% of the variance in positive affect (see Table 3). Perceived stress and social support emerged as significant predictors in the model. Adding the four types of coping strategies into the model in the second step of the regression improved its ability to explain variance in positive affect significantly, with the equation able to explain 61% of the variance (ΔR^2^ = .11, *p* < .001). The overall regression was statistically significant, *F*(8, 288) = 21.51, *p* < .001. Collective coping, avoidance coping, social support, heritage acculturation and Canadian acculturation did not provide a significant contribution to predicting positive affect. Engagement coping positively predicted positive affect, while perceived stress and religious coping emerged as negative predictors (see Table 3).

**Table 3:** **Regression models predicting positive affect**

		B	SE B	β	R^2^	Adj R^2^	Δ R^2^
Model 1					.269***	.259***	
	Perceived stress	-.47***	.052	-.46***			
	Social support	.09**	.033	.14**			
	Canadian acculturation	.05	.033	.07			
	Heritage acculturation	.02	.026	.04			
Model 2					.374***	.357***	.105***
	Perceived stress	-.35***	.054	-.34***			
	Social support	.06	.032	.09			
	Canadian acculturation	.04	.031	.06			
	Heritage acculturation	.00	.026	.01			
	Religious coping	-.27***	.063	-.21***			
	Collective coping	-.01	.051	-.01			
	Avoidance coping	-.07	.056	-.06			
	Engagement coping	.31***	.064	.26***			

Note: **p < .01,***p < .001.

### Hypothesis 2 (regression model for negative affect)

In the first step of the regression model predicting negative affect, perceived stress, social support, Canadian acculturation and heritage acculturation, as the control variables, significantly predicted the equation, *F*(4, 292) = 39.42, *p* < .001 (see Table 4). These variables combined explained 60% of the variance of negative affect. Only perceived stress emerged as significant predictor in this equation. Adding the coping variables into the equation in the second step of the regression significantly increased its ability to predict negative affect (ΔR^2^ = .03, *p* < .05). This overall equation also significantly predicted negative affect, *F*(8, 288) = 21.71, *p* < .001, accounting for 61% of the total variance. Perceived stress and avoidance coping were found to positively predict negative affect, while no other variables emerged as significant predictors (see Table 4).

**Table 4:** **Regression models predicting negative affect**

		B	SE B	β	R^2^	Adj R^2^	Δ R^2^
Model 1					.35***	.34***	
	Perceived stress	.55***	.045	.59***			
	Social support	.01	.029	.01			
	Canadian acculturation	.00	.028	.01			
	Heritage acculturation	.02	.023	.05			
Model 2					.38***	.36***	.026*
	Perceived stress	.51***	.050	.54***			
	Social support	.01	.030	.02			
	Canadian acculturation	.01	.029	.02			
	Heritage acculturation	.01	.024	.03			
	Religious coping	-.00	.058	-.00			
	Collective coping	.03	.048	.04			
	Avoidance coping	.17***	.052	.16***			
	Engagement coping	-.03	.059	-.03			

Note: *p < .05, ***p < .001.

## Discussion

The central purpose of the current study was to test hypothesized models of positive and negative affect in a multiethnic undergraduate sample of students in Canada using theoretically guided psychosocial and cultural variables as predictors. As such, the study is among the first to empirically examine the critical relationships among stress, social support, bidirectional acculturation, religious cooing, cultural coping, positive affect and negative affect simultaneously through regression analyses. A number of key findings have been revealed in this research and are reviewed below.

Hypothesis 1 was supported by the results of the regression analysis as the overall proposed predictive model of positive affect was found to be statistically significant. The eight psychosocial, acculturation and coping variables collectively accounted for 61% of the variance in positive affect, indicating a large effect size (Cohen 1988). Hence, the results suggest that the quality of positive emotions experienced by the undergraduate participants, which included the feelings of interest, excitement, strength, pride, alertness, inspiration, etc., were related to their perception of their stress level, degree of social support, cultural orientation/identification as well as the kind of coping strategies they used to deal with academic stressors, such as having academic difficulties as depicted in the scenario in the Cross-Cultural Coping Scale. These variables, therefore, represent both risk and protective factors for positive emotional well-being in the sample.

The finding of the regression analysis that perceived stress was a negative predictor of positive affect in the current study is well supported by previous research with other sample populations, including the study of caregivers of cancer patients by Fitzell and Pakenham (2010) and the study of Chinese international students by Pan et al. (2008) reviewed earlier. It is intuitive that experiencing high level of stress of any kind would inevitably inhibit or tax an individual’s reserve of positive emotions and resources. Similarly, the present regression results showed that the use of religious coping strategies by the participants was associated with reduced positive emotions. That is, religious coping may have adverse effect on the positive emotional well-being for university students. Interestingly, this finding is not consistent with other research which suggests that a potential positive and favorable relationship exists between religion/spiritually and individuals’ health outcomes (Pargament 1997; Hill and Pargament 2003).

One possible explanation is that the typical stressors experienced by university/college student maybe more pragmatic in nature, such as having to achieve good grades and completing academic assignments. Therefore, coping through religious means in attending to these issues may not be effective or even useful in bringing about emotional relief for university students. Instead, these stressors may call for more tangible and instrumental coping strategies on the part of the students (Lazarus and Folkman 1984). Indeed, this postulation finds support in the fact that the regression results also showed that engagement coping was the only other cultural coping strategies to significantly predict positive affect in a positive direction in the current sample. This relationship indicates that the use of problem-focused and action-oriented coping strategies, such as engagement coping, facilitates the promotion of positive emotions among the participants in the midst of dealing with stress. This outcome aligns with the assertion made by Folkman and her colleagues, which links problem-focus coping to positive affect (Folkman 1984; Lazarus and Folkman 1984).

Hypothesis 2, which stated that the proposed model based on the eight psychosocial, acculturation and coping predictor variables would significantly predicted negative affect in the sample, was also supported by the results of the regression analysis. Paralleling the previous finding for the model on positive affect, these eight predictor variables also collectively accounted for 61% of the variance in negative affect with a large effect size (Cohen 1988). Again, the participants’ appraisal of stress level, available social support, level of acculturation and cultural identification, and endorsement of various coping strategies had an impact on their overall negative emotional experiences, including feeling of guilt, shame, irritability, distress, nervousness, hostility, etc. In other words, these factors taken together provide a good explanatory model for current participants’ negative emotions.

In particular, greater perceptions of stress on the part of participants significantly contributed to a higher level of negative affect. This close positive association between life stress and strain and negative affect has been frequently reported in many previous studies (e.g., Green et al. 2012; Nelson 1990; Paukert et al. 2006). In addition, avoidance coping behaviors were also found to be a significant predictor of negative affect in a positive direction in the current sample – those who utilized more avoidance behaviors reported more negative emotions. Conceptually, this finding is in accordance with the theoretical prediction of Folkman’s (1984) coping theory, which postulates an interwoven relationship between negative emotion and emotion-focused coping behaviors, including avoidance coping. This finding is also consistent with observations made in other cross-cultural coping studies. Avoidance coping has been shown to be maladaptive and relates to negative psychological and emotional outcomes for ethnic minorities across different stressors, including male gender role conflicts (Wester et al. 2006) and racial discrimination (Noh and Kaspar 2003) as examples.

Unexpectedly, social support was not found to be a significant predictor in the regression analyses of either positive or negative affect. This is despite the fact that previous studies of affective quality have found social support to be a relatively robust predictor of affects (e.g., Fitzell and Pakenham 2010; Green et al. 2012). While the exact reason for this finding in the current undergraduate sample is not clear, it is possible that the issue might stem from the characteristics of the social support measure used in the present research. For example, a previous study by Fitzell and Pakenham (2010) assessed participants’ social support in terms of both ‘availability’ and ‘satisfaction’ of support in predicting positive affect. The measure used in the current study, the Multidimensional Scale of Perceived Social Support (Zimet et al. 1988), however, assessed only the perceived ‘availability’ of social support by the participants. To help clarify this issue future research should employ social support scales with more nuanced dimensionalities in predicting affective quality in a similar sample population.

Finally, in addition to the findings of the main model-testing regression analyses, the present study also identified a number of ancillary findings worth noting. First, in the present sample the correlation between positive and negative affect was *r* = −.27, *p* < .001. Such a correlation suggests that the two affective constructs were only moderately related with a medium effect size according to Cohen (1988). The strength of the correlation in the current study is comparable to those reported in the original instrument development and validation study of the PANAS by Watson et al. (1988). It was also evident from this study that the patterns of predictors for positive affect vs. negative affect were clearly distinguished from each other. This finding further underscores the relative independence of the two affects. Thus, the current results lend support to the Two-Factor theory of affect (Diener and Emmons 1985) and suggest the generalizability of such a theory to the current multiethnic university sample.

Although neither heritage acculturation nor Canadian acculturation provided significant explanatory ability to the regression equations, some interesting findings did emerge. In the present study heritage acculturation and Canadian acculturation were not statistically correlated. This provides additional evidence for the bidirectional model of acculturation (i.e., viewing cultural identification with one’s own or one’s parents’ culture of origin independently from cultural identification with the dominant society) (Ryder et al. 2000). It is notable that even though neither heritage acculturation nor Canadian acculturation significantly predicted either of the affects, both types of cultural orientation formed positive relationships with both positive and negative affect. This suggests that positive and negative affect form independent relationships with acculturation and further highlight the orthogonality between the two sets of affects.

Interestingly, higher levels of heritage acculturation were found to be significantly correlated with less use of religious coping but more use of collective coping. This specific relationship between heritage acculturation and collective coping is analogous to the finding of an earlier coping study of Chinese Canadian adolescents (Kuo et al. 2006). Using the same coping scale as in the current research, the Cross-Cultural Coping Scale, the study showed that Chinese Canadian adolescents who identified more strongly with Canadian cultural values reported less collective coping in dealing with culture-related stressors (i.e., intergenerational conflict and racial discrimination) than those who had a weaker identification with Canadian cultural values. The finding provides further clues to the association between the use of collective coping strategies and the characteristics of individuals’ cultural orientations (Kuo 2011). It is posited that undergraduate students who adhered more to their culture of origin or culture of their parents would likely to have held stronger collectivistic values than those who did not. Consequently, for these students their collectivism is extended or manifested through a greater tendency to endorse collective coping, through group-, relationally-, and culture norm- based stress responses (Kuo 2013).

### Limitations of the study

It is important to consider the results of the present investigation in light of a number of limitations associated with the characteristics of the present research. First, the study was based on a cross-sectional design. Consequently, any causal relationships between the psychosocial, acculturation, and coping variables in the predictor equations and positive and negative affect can only be inferred. The interpretation of the findings, therefore, requires extra caution. Second, the current results were established on a sample that was predominantly women (76%), due to the fact that the majority of the participants were recruited from the research participant pool among students who took psychology courses at the university where the study occurred. It is unclear whether and to what extent this gender disproportion among the participants might have affected the study's results. Undoubtedly, future studies examining positive and negative affect should either seek more balanced gender representations in a study or focus on the experiences of either men or women separately. Lastly, the present investigation was conducted with university student sample in a single university in Southwestern Ontario, Canada. The generalizability of the research findings for student populations elsewhere or nonstudent populations cannot be verified until future research.

### Implications and directions for future research

In view of the scarcity of existing cultural and cross-cultural research on affects in general and the limitations of the present study noted above, there is significant potential for future research in this area. First, future studies involving prediction of positive and negative affect would benefit from having a longitudinal design. Such a methodological approach would enable researchers to more precisely disentangle any causal relationship between the psychosocial, cultural, and coping predictors and the affects. Second, in the current study neither heritage acculturation nor Canadian acculturation significantly predicted positive or negative affect in the regression analyses. While acculturation was not a primary focus of this investigation, it was nevertheless unclear whether these findings were an artifact of the specific characteristics of the current sample, with nearly 30% of the participants being of White/European background and 42.5% of them coming from second and later generation immigrant background. To verify these observations future research should seek to replicate the present investigation with sample populations for which acculturation and cultural adjustment experiences may be more salient and relevant (e.g., newcomer immigrants, international students, sojourners, etc.).

Third, it is observed that the prevailing cross-cultural literature on acculturation is currently dominated by research that views and assesses cultural adaptation/adjustment from a narrow psychological distress and symptomology (depressive and anxiety symptoms, etc.) perspective (Yoon et al. 2012). The current study, however, demonstrated the benefits of exploring the potential positive side of stress-coping and cultural adaptation empirically – an approach that would afford the field a fuller, richer, and more comprehensive understanding of coping, adjustment and well-being among culturally diverse individuals (Folkman 2008; Pan et al. 2008). Hence, it is hoped that this present study can serve as a catalyst to encourage other researchers in cultural/cross-cultural psychology and acculturation to consider incorporating positive emotions and positive indicators of adjustment (e.g., subjective well-being and resilience) into future research.

Finally, an attempt was made in the present study to search and piece together various discrete yet relevant theoretical perspectives to help guide and inform the research (as reviewed under the “Theoretical frameworks” section in the introduction). From this standpoint, it seems that continuous theory development works in this area would be timely and highly desirable. More concerted and systematic efforts are needed to articulate and integrate contemporary theories of stress-coping-emotion, acculturation, and psychosocial predictive models of positive and negative affect. Ultimately, scholars should strive towards extending existing acculturation theories and propositions to a framework of stress-coping-adaptation that is informed and enriched by the emerging theories of affects and emotions.
